# Proteomics in Host-Protozoan Parasite Interactions and Development of Drug and Vaccine

**DOI:** 10.34172/apb.2021.024

**Published:** 2020-07-15

**Authors:** Awanish Kumar

**Affiliations:** Department of Biotechnology, National Institute of Technology Raipur, Raipur – 492010, Chhattisgarh (INDIA).

## Dear Editor,

Current control measures of protozoan infection rely only on chemotherapy to improve the disease. Vector control is also doing to reduce the transmission of protozoan infections. To date, there are no reliable vaccines are available against these infections and there is also an alarming rise of drug resistance.^1^ However, in the longer term, there is a consensus that vaccines could become a key prophylactic measure in the control of the diseases caused by protozoan parasites *Leishmania* (leishmaniasis), *Plasmodium* (malaria), and *Trypanosoma* (trypanosomiasis). Unfortunately, the vaccine development has been hindered by major antigenic diversity in protozoan parasite and they have also a digenetic or multigenic life-cycle in two hosts (human and vector) at least. Contribution of the target host population genetics and their susceptibility to infection/disease is an equally important concern for the design and accomplishment of anti-parasitic vaccines.

Rhoptry organelles are part of the defining features of the phylum Apicomplexa (*Plasmodium*). The contents/function of these rhoptries have been the focus of substantial attention to comprehend the ability to invade a host cell which is most important of all apicomplexan characteristics.^2^ The obligate intracellular parasites Trypanosoma can parasitize a wide variety of cells during which these specialized secretory organelles play a central role.^3^ Parasitophorous vacuole facilitates the survival of the *Leishmania* parasite in humans.^4^ Genome sequence availability to support a proteomic database represents an extraordinary opportunity to characterize the composition and function of the protein in protozoan parasites. The rapid expansion of our understanding in proteomics has directed to new understandings into their function and set the foundation for a more complete insight of how these parasites contribute to invasion and intracellular survival process in the host.^5^ Immune responses to protozoan infection are highly multifaceted and it involves both humoral and cell-mediated responses to parasitic antigens array. In these protozoan parasites, infection often involves multiple lifecycle stages, which enhances antigenic diversity. Recent efforts have intensive to evaluate the response of defined parasite T-cell epitopes to examine the molecular basis of the immune reaction prompted during the infection with protozoan parasites. The severity of infection is a function of the infecting species together with consequent inflammatory/immune responses and host genetics. It has shown by the genetic and immunologic studies using the animal model and now becomes evident. They seem to singularly involve in immune subversion and require the participation of both B-cells and CD4^+^ T-cells during pathogenesis. A low level of IFN-γ production in host appears to be an important feature of disease progression. The monocyte recruitment and antigen presentation at the site of infection appear to be perilous for the pathogenesis development of the protozoan parasite. The roles of immune B-cells and T-cells in pathology could affect the efficiency of vaccines against protozoan parasite-mediated infectious diseases.

The proteome is the collection of all the proteins produced from the commands of genetic material in the cell. Proteomics gives a profile of every protein that is being synthesized by a cell. Because of the accessibility of the relevant and advanced proteomics technology, the emphasis has been given to identifying novel target proteins that could serve as a potent drug target or vaccine candidate. Proteins establish the biological phenotype of an individual therefore; proteins are the most important targets for the therapeutic agents. The no. of therapeutic agents all infectious disease is very least, and it is probably due to the shortage of drug targets. A year-wise list of nos. of drugs approved by the Food and Drug Administration (FDA, USA) for sale in the market against infections and infectious diseases is listed in Table 1. This table gives the idea of sluggish growth of drug development against infectious diseases. Due to a shortage of target protein in protozoan parasite, very less no. of new molecules is deciphered against the infection. We must speed up the process and proteomics in this direction to extract important protein from the proteome pool of parasite that would be an added advantage. Proteomics can be used to identify new targets proteins in the case of protozoan parasites that are unexplored to date. It would further pave the way for the development of new drug or effective vaccines against disease caused by protozoan parasites. Additionally, proteomics also gives information about virulent proteins post-translational modifications (PTMs) (such as acylation, glycosylation, methylation, phosphorylation, etc). PTMs give vital information about the functioning of many proteins and it may recognize as the most important mechanism in the cellular regulation of pathogen. Proteomics is now widely employed in the study of parasite proteome. Therefore, proteomic analysis imprisonments all these changes and may provide exclusive and valuable information in host-protozoan parasite interactions for the development of drug/vaccine against infection caused by a protozoan parasite.These proteomic techniques may be applied to human protozoan pathogens to identify novel protein targets for the development of potent drug or vaccine candidates.

**Table 1:** Year-wise number of anti-infectious agent approved by the FDA^6^

**Year**	**No. of anti-infectious agents approved by the FDA, USA**
2019	06
2018	14
2017	13
2016	07
2015	09
2014	13
2013	07
2012	13
2011	09
2010	04
2009	04
2008	05
2007	04
2006	05
2005	02
2004	00
2003	04
2002	08
2001	13
2000	09
1999	04
1998	05
1997	22
1996	22
1995	08

## Conclusion

Many laboratories across the globe are utilizing proteomics as rapidly emerging technology to elucidate the fundamental mechanism associated with the severity detection of disease, cancer development, drug development, and identification of disease conditions in patients.^7^ Despite some challenges, proteomics has rapidly extended to comprise the finding of novel targets and biomarkers for a vast clinical application like early detection, diagnosis, and prognosis. The pharmaceutical application of proteomics could be explored in-depth for the documentation of novel drug targets. Several innovative proteomics technologies (two-dimensional polyacrylamide gel electrophoresis maps, SELDI, MALDI, multidimensional protein identification technology (Mud-PIT), protein array techniques, isotope-coded affinity tag, NMR spectroscopy, X-ray crystallography, and computational methods such as *de novo*/comparative prediction of protein structure, molecular dynamics simulation, etc) have progressed to achieve these goals. These proteomics technologies have been being used in different combinations. Proteomics has been extensively used to develop protein databases for protozoan parasite and evaluate the expression profile of genes under different environmental conditions. Such kind of proteomic investigation provides access to global changes related to specific mutations and explain drug targets of disease-causing protozoic pathogens. Above mentioned proteomics techniques may also be used to identify B-cell and T-cell antigens response of host after parasitic infection within complex protein mixtures when they coupled to immunological assays. This article provides a glimpse of the proteomics field and confers the extensive use of proteomic technologies to researchers. Article accentuates the proteomic approaches application in the development of a strong understanding of host-protozoan parasite interactions and promising drug targets and vaccines against infection of protozoa parasite.

## Ethical Issues

Not applicable.

## Conflict of Interest

The author declares no conflict of interest.

## Acknowledgments

Author thanks the National Institute of Technology, Raipur (CG), India for continuous support and assistance during research work and scientific writing.
